# A multifunctional DNA nano-scorpion for highly efficient targeted delivery of mRNA therapeutics

**DOI:** 10.1038/s41598-018-28542-3

**Published:** 2018-07-05

**Authors:** Dandan Li, Fei Mo, Jiangling Wu, Yong Huang, Huihao Zhou, Shijia Ding, Weixian Chen

**Affiliations:** 1 Department of Laboratory Medicine, The Second Affiliated Hospital of Chongqing Medical University, Chongqing, 400010 P.R. China; 2 Key Laboratory of Clinical Laboratory Diagnostics (Ministry of Education), College of Laboratory Medicine, Chongqing Medical University, Chongqing, 400016 P.R. China

## Abstract

The highly efficient cancer cell targeted delivery plays an important role in precise targeted therapies. Herein, a multifunctional DNA nano-scorpion nanostructure (termed AptDzy-DNS) functioned with aptamers and DNAzyme is developed for highly efficient targeted delivery of mRNA therapeutics in gene therapy. The designed AptDzy-DNS is self-assembled with specific aptamers as “scorpion stingers” for targeting tumor cell and DNAzymes as “scorpion pincers” for targeted gene therapy by cleaving mRNA into fragments. The as-prepared AptDzy-DNS can effectively distinguish cancer cells from normal cells by specific cross-talking between aptamers on AptDzy-DNS and overexpressed cell-surface receptors. In the process of gene therapy, by reacting with Mg^2+^-dependent DNAzyme on AptDzy-DNS, the mRNA oligonucleotide in cancer cell is auto-cleaved into broken strand, failing to be translated into corresponding protein. Following, the downregulation protein can block cancer cell growth and realize highly efficient targeted therapies. The results demonstrate that the multifunctional AptDzy-DNS shows promise for targeted cancer cell discrimination, highly efficient targeted delivery of mRNA therapeutics in gene therapy. Thus, this developed strategy provides impressive improvement on gene targeted therapy and paves the way for application of AptDzy-DNS in human cancer targeted therapies.

## Introduction

Targeted gene therapies that selectively block gene amplification and overexpression by delivery therapeutic reagents in mammalian cells are emerging promising approaches for the precise treatment of human cancer diseases^1–3^. An ideal targeted gene therapy approach should specifically target cancer cells, aim to suppress molecular pathways that are critical for cancer cell growth and maintenance, and minimize the non-specific killing of normal cells as well as realize highly efficient targeted therapies^4–6^. Recently, mRNA therapeutics including deoxyribozymes (DNAzymes)^7–9^, small interference RNA (siRNA)^10^ and antisense oligonucleotides^11^, have been developed as potential clinical therapeutic reagents for oncogene silence or downregulation. Among them, siRNA-based gene therapy technology encounters a key challenge of its delivery efficiency and off-target effect, which greatly limits its potential practical application. DNAzymes, such as 10–23 and 8–17 DNAzyme consisting of a catalytic core and two recognition arms flanked, can be designed to hybridize with complementary sequences in a target messenger RNA (mRNA) and then cleave mRNA at predetermined phosphodiester linkages. Compared to siRNA-based technology, DNAzymes hold added capacities of low-cost synthesis, high selectivity, and significant catalytic efficiency as well as free of off-target effect^12^. Thus, engineering a DNAzyme-based gene inactivation or downregulation strategy is becoming a promising alternative for highly efficient targeted gene therapy. In spite of the increasing interest in DNAzyme-mediated gene silencing as a therapeutic strategy, delivery specificity and efficiency still remain the major hurdles to conquer for practical applications due to physiological barriers of cell membranes and degradative enzymes.

To solve these issues, many kinds of materials have been explored as delivery carriers for gene therapy, including liposomes, cationic polyelectrolytes, and inorganic nanoparticle^13–17^. Though these targeted delivery methods have showed some merits, they still suffer from some drawbacks in practical applications, such as less cell-specific manner, complex surface modification process, low loading efficiency, and the damage of immunogenic response or toxicity^18,19^. Thus, the restricted efficiency and specificity remain the critical barriers hindering the broad clinical application of the above-mentioned methods for precise targeted therapies^20,21^. Taking advantages of Watson-Crick base-pairing, self-assembled DNA nanostructures can offer the advantages of flexible design, controllable size and orientation, ease of bioconjugation and excellent biocompatibility, and demonstrate potential application in targeted therapies^22–24^. Recently, numerous DNA nanostructures^25,26^, such as DNA nanotubes^27^ and DNA tetrahedrons^28^ have been attracting wide attention for precise targeted therapies. However, only few works about the incorporation of internalizing DNAzymes into DNA nanostructure for targeted gene therapies have been reported to date. Thus, the further development and improvement of DNAzymes-DNA nanostructures in practical application is still in urgent need.

**Figure 1:**
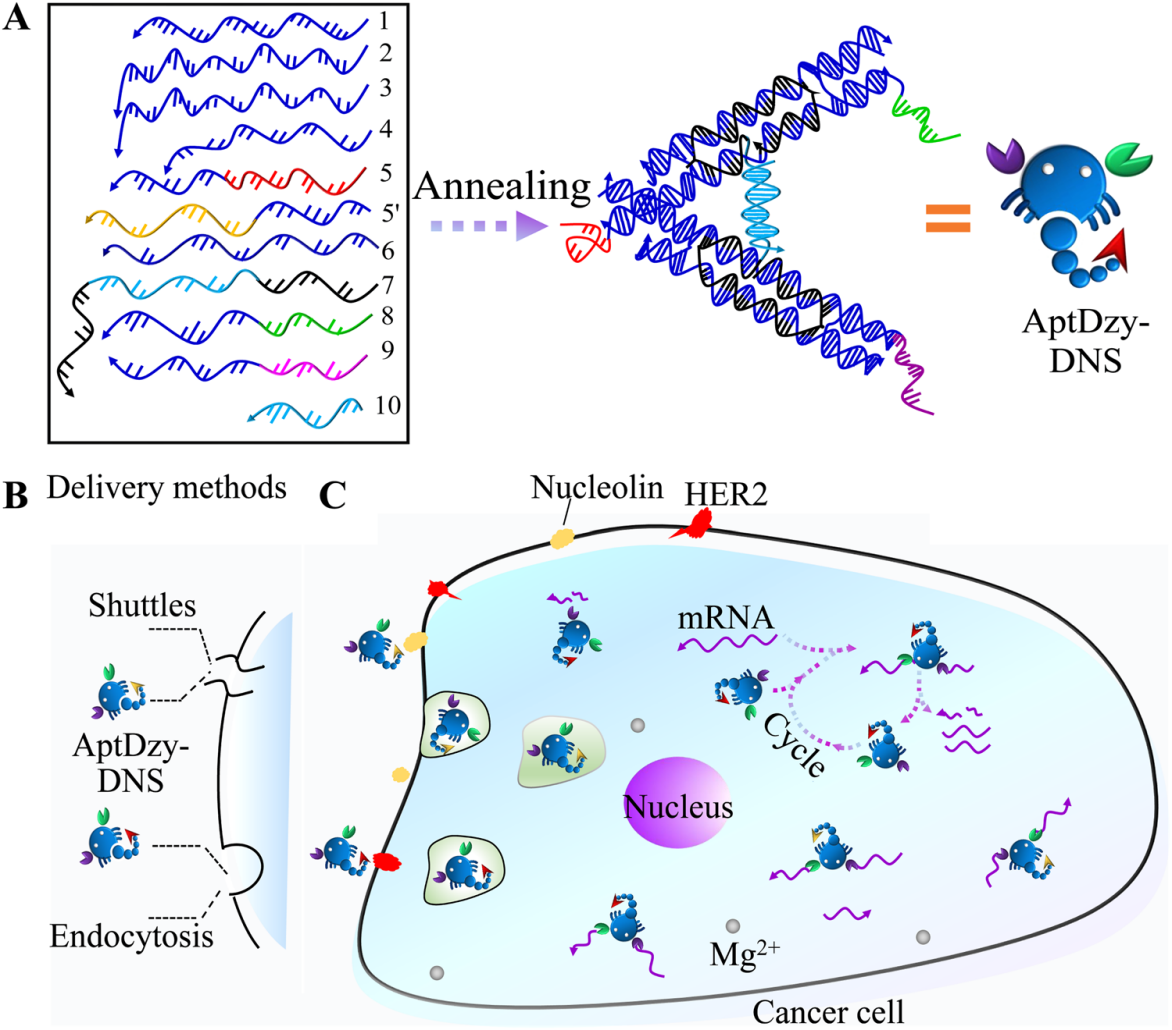
(**A**) Assembly of AptDzy-DNS (**B**) Targeted delivery methods. (**C**) Schematic illustration of AptDzy-DNS mediated highly efficient gene therapy.

Using human epidermal growth factor receptor (*HER2*) mRNA as a target gene^29–32^ and SK-BR-3 (breast cancer cell) as an *in vitro* model system, this work developed a self-assembled scorpion-like DNA nanostructure (termed AptDzy-DNS) that functioned with aptamer and DNAzyme for highly efficient targeted gene therapy (Fig. 1). Briefly, the AptDzy-DNS mainly consists of specific aptamers as “scorpion stingers” for targeting tumor cell and DNAzymes as “scorpion pincers” for cleaving mRNA into pieces. In light of surface receptors expressed on tumor cells^33–35^, the specific aptamer is self-assembled in AptDzy-DNS to guarantee the highly efficient and specific cell targeting. Upon the smart design of the DNA sequences, DNAzymes on AptDzy-DNS can bind to apoptosis-related mRNA and cleave it at predetermined phosphodiester linkages, leading to downregulation of its corresponding protein and thus inhibition of cancer cell proliferation. This developed AptDzy-DNS is proved to be a simple and highly efficient platform for therapeutic reagents delivery and targeted gene therapy with good biocompatibility and no side effects.

## Results and Discussion

### Principle of multifunctional AptDzy-DNS mediated gene therapy

The AptDzy-DNS was constructed by self-assembly of oligonucleotides (Fig. 1A), which mainly consists of specific aptamers as “scorpion stingers” for targeting tumor cell and DNAzymes as “scorpion pincers” for gene silencing by cleaving mRNA into pieces. With the aid of receptors on cells-surface,the specific anti-receptor aptamer self-assembled on AptDzy-DNScan ensure the specific cell targeting delivery by cross-talking of scorpion stingers and overexpressed cell-surface receptors. To enhance the delivery efficiency of AptDzy-DNS, dual cancer cell-surface overexpressed receptors (nucleolin and HER2) are simultaneously used to increase the local concentration of receptors on cell membranes. Sinceanti-nucleolin aptamer (NApt) and anti-HER2 aptamer (HAp) are assembled on AptDzy-DNS, the AptDzy-DNS can conveniently internalize in cells by cell surface receptors-mediated shuttling or endocytosis (Fig. 1B). In the process of gene silencing (Fig 1C), two scorpion pincers at the end of AptDzy-DNS can hybridize with HER2 mRNA to form stable active DNAzymes and cleave it at predetermined phosphodiester linkages in the presence of cellular Mg^2+^, resulting in cleavage of HER2 mRNA with the aid of Mg^2+^ -dependent DNAzymes and release of AptDzy-DNS from the substrate strand of *HER2* mRNA. The released AptDzy-DNS could bind to another intact *HER2* mRNA, leading to continuous cleavage of apoptosisrelated *HER2* mRNA, which could in turn downregulate the corresponding protein expression and inhibit cancer cell proliferation. Thus, the targeted gene therapy was successfully achieved with minimal adverse effects on normal cells by silencing or downregulating HER2 mRNA. The results demonstrate that the multifunctional AptDzy-DNS shows promise for targeted cancer cell discrimination, and highly efficient gene therapy.

### Characterization of multifunctional AptDzy-DNS

**Figure 2:**
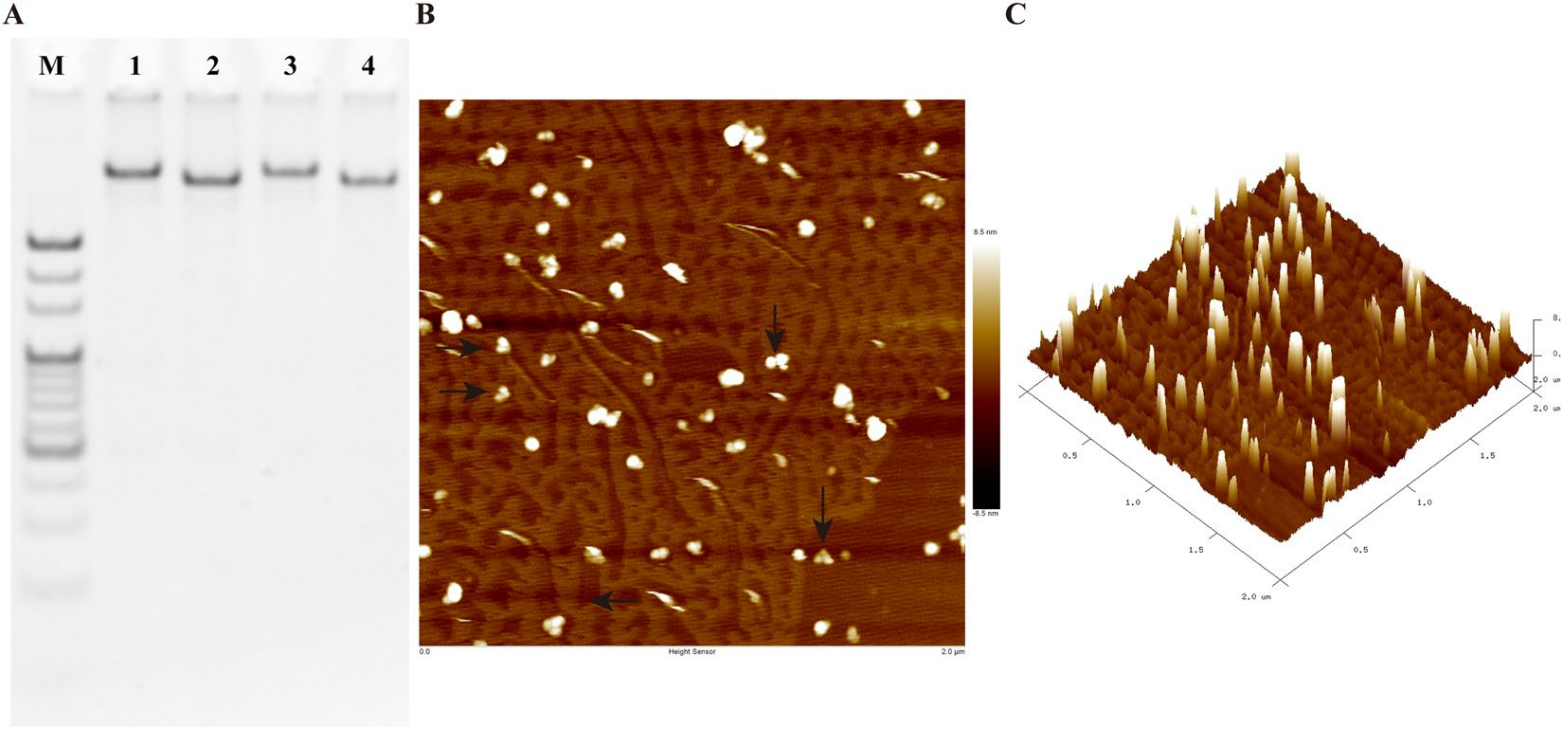
Characterization of AptDzy-DNS self-assembly. (**A**) PAGE analysis of AptDzy-DNS self-assembly. Lanes 1-2 represent closed AptDzy-DNS and open AptDzy-DNS, Lanes 3-4 represent AptDzy-DNS incubated in serum for 2 h, and DNA ladder marker. (**B**,**C**) AFM image of AptDzy-DNS. Phase image, the scale bar is 300 nm.

The multifunctional AptDzy-DNS was synthesized and improved according to our previous work^36^. According to the helical diameter and distance between two base pairs in a double-stranded DNA, for the open AptDzy-DNS, the edge length and the end of arms of the constructed nanostructures can be calculated as ~20.06 nm and ~16 nm, respectively. For the closed state, the end of arms of is ~6.9 nm, according to Förster resonanceenergy transfer measurements (FRET)^37,38^. The assembly of AptDzyDNS was characterized by 6% polyacrylamide gel electrophoresis (PAGE) experiment (Fig. 2A). Upon mixing ten oligonucleotides, the mixture showed a distinct bright band of DNA with more than 500 bases ((Fig. 2A lane 1), indicating the successful synthesis of single DNS containing DNA 1–9 and 10 (set strand). For the closed state, the mixture without set strand produced a single band with less bases that migrated much faster than band in lane 1 (Fig. 2A lane 2). Meanwhile, the AptDzy-DNS could maintain structural stability in serum over 2 h (Fig. 2A lane 3 and lane 4). The AptDzy-DNS structure was also confirmed by atomic force microscopy (AFM). As shown in Fig. 2B,C, the DNA nanostructures appeared to be quite uniform, rigid and well-dispersed (AptDzy-DNS, indicated by arrows).

### The feasibility of cleavage activity of AptDzy-DNS *in vitro*

**Figure 3:**
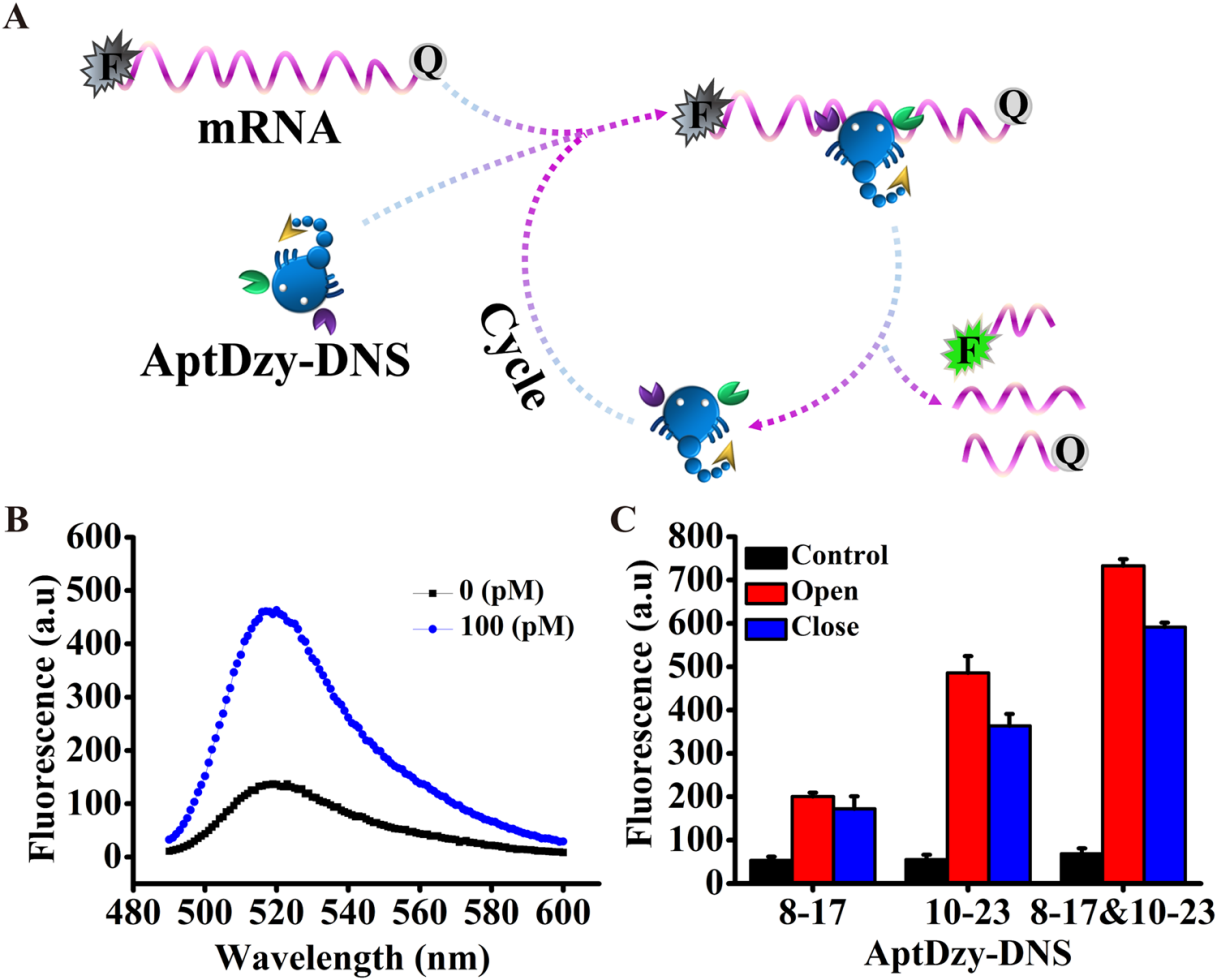
*In vitro* cleavage activity of AptDzy-DNS. (**A**) Schematic representation of the designed AptDzy-DNS for nicking mRNA. (**B**) Fluorescence spectra of AptDzy-DNS mixed with mRNA at 0 and 100 pM. (**C**) Comparison of the cleavage activity of 10–23 and 8–17 AptDzy-DNS.

The feasibility of AptDzy-DNS mediated the digestion of *HER2* mRNA was verified with FRET (Fig. 3) by mixing the synthetic *HER2* mRNA with AptDzy-DNS in buffer. Here, *HER2* mRNA was tagged with fluorescein isothiocyanate (FITC) and black hole quencher (BHQ1) at the 5′-end and 3′-end, respectively (Fig. 3A). As shown in Fig. 3A, in the presence of *HER2* mRNA, two overhangs at the end of AptDzy-DNS hybridized with *HER2* mRNA to form stable active DNAzymes, resulting in cleavage of *HER2* mRNA with the aid of Mg^2+^-dependent DNAzymes. The fracture of *HER2* mRNA enabled AptDzy-DNS to hybridize with another *HER2* mRNA, leading to continuous removal of BHQ1 away from FITC. Thus, the fluorescence recovery of FITC from cleaved sequence was significantly enhanced. On the contrary, without hybridization of AptDzy-DNS and *HER2* mRNA, there was no observable cleavage occurred (Fig. 3B). As shown in Fig. 3C, opened AptDzy-DNS demonstrated strong fluorescence intensity than that of closed AptDzy-DNS, suggesting that opened AptDzy-DNS was more beneficial for digestion of *HER2* mRNA due to the decreased steric hindrance. In addition, the fluorescence intensity was much stronger when 10–23 and 8–17 DNAzyme were simultaneously overhanging at each arm of AptDzy-DNS than that of only one kind of DNAzyme. This phenomenon might attribute to that 10–23 and 8–17 DNAzyme offer more binding domains for mRNA, which can decrease incomplete hybridization with single mRNA due to increased local concentration of DNAzyme.

### Optimization of experimental conditions

To achieve the excellent assay performance, some experiment conditions were optimized with the synthetic *HER2* mRNA at 100 pM. Because Mg^2+^ plays an important role in cellular microenvironment, the concentration of Mg^2+^ was firstly optimized. As shown in Supporting Information, Fig. S1, the fluorescence intensity increased dramatically with the increasing amount of Mg^2+^ and kept constant over the concentration of 1.6 mM (Fig. S1A,B), which is close to that of cellular microenvironment. Therefore, 1.6 mM of Mg^2+^ was used in the following *in vitro* experiments. Next, the effect of their action time was also evaluated in Fig. S1C, the maximum fluorescence intensity value was achieved at 30 min, suggesting that the developed AptDzy-DNS mediated gene therapy holds the capacity of time-saving and significant nicking efficiency.

### Feasibility of AptDzy-DNS induced translocation

**Figure 4:**
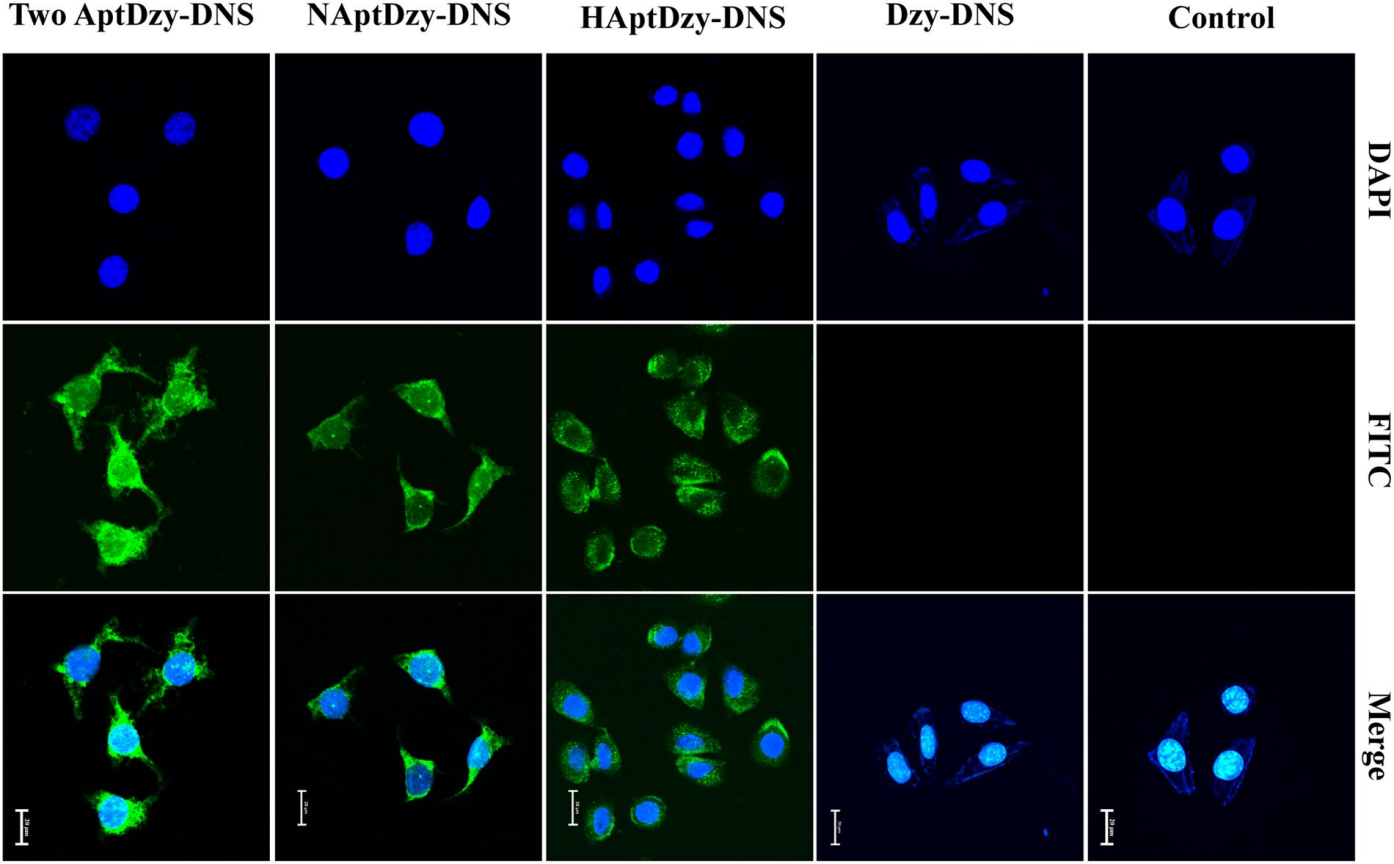
Characterization of AptDzy-DNS induced translocation. Confocal microscopy images of SK-BR-3 cells after incubation with 0.5 μM Dzy-DNS without aptamer, HAptDzy-DNS, NAptDzy-DNS, and the mixture of HAptDzy-DNS and NApt-Dzy-DNS for 2 h. Scale bars: 20 μm. (Note: NApt, anti-nucleolin aptamer; HApt, anti-HER2 aptamer; Apt, aptamer).

First, the delivery efficiency of AptDzy-DNS was investigated by comparing cellular fluorescence of FITC-AptDzy-DNS with aptamer vs without aptamer. Confocal fluorescence microscopy images showed that FITC signals from FITC-AptDzy-DNS with aptamer in SK-BR-3 cells were much higher than those of Dzy-DNS without aptamer at the same FITC-AptDzy-DNS concentration and integration time (Fig. 4). In addition, the signal distribution of FITC-NAptDzy-DNS was distinct within both cytoplasm and nucleus due to the shuttle capability of nucleolin between cytoplasm and nucleus. Compared to FITC-NAptDzy-DNS, the fluorescence signal distribution of FITC-HAptDzy-DNS was obvious within cytoplasm, where the FITC-HAptDzy-DNS appeared in clusters.

**Figure 5:**
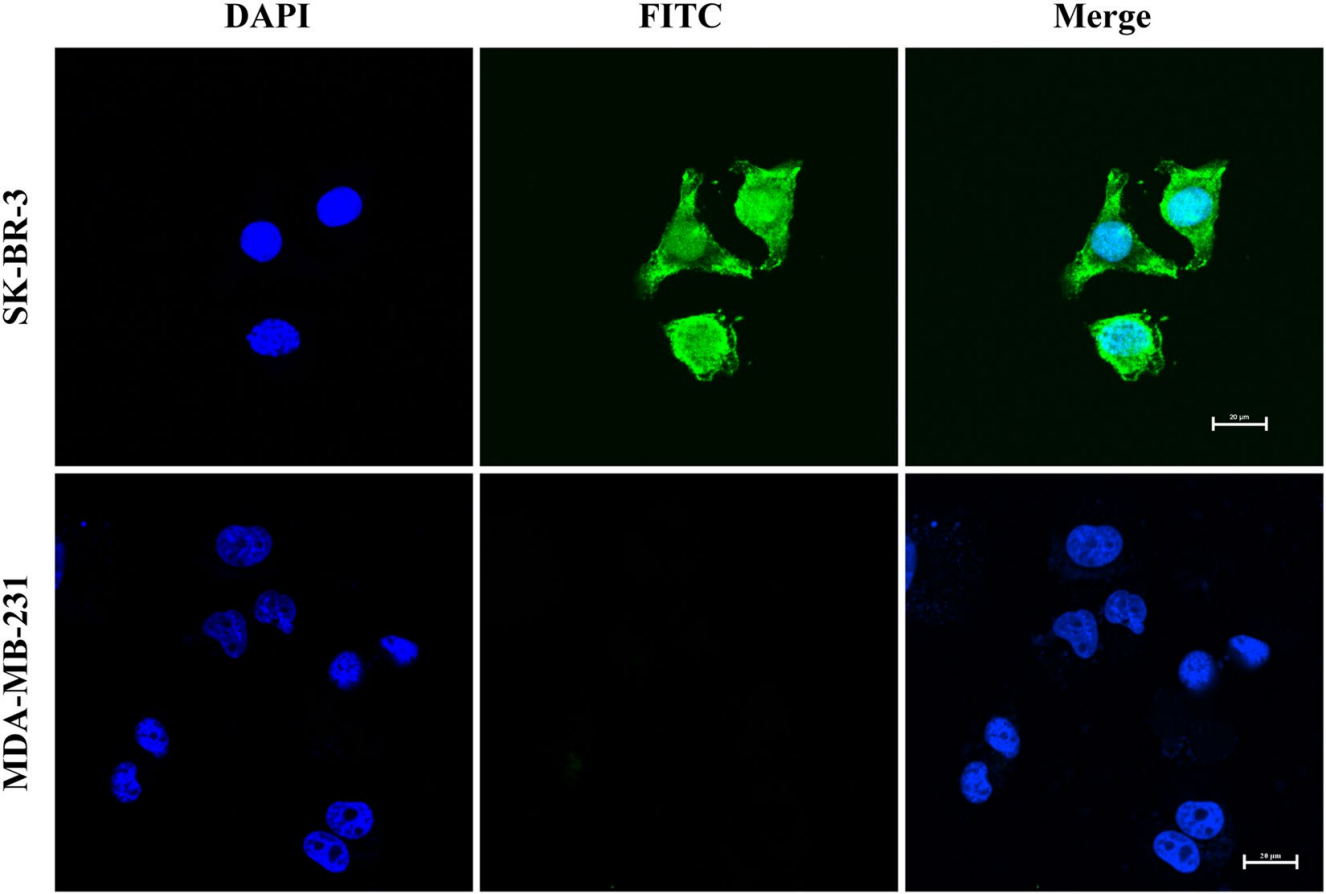
Specificity of AptDzy-DNS induced translocation. Confocal microscopy images of SK-BR-3 cells and MDA-MB-231 cells after incubation with 0.5 μM AptDzy-DNS for 2 h. Scale bars: 20 μm.

Next, the integration time was examined via confocal fluorescence microscopic and flow cytometry for confirming the efficiency of AptDzy-DNS induced cellular translocation (Figs S2 and S3). The FITC fluorescence increased with the increasing time and kept constant after 2 h, which was shorter than previous reported work^37^, suggesting that the translocation performance of AptDzy-DNS was efficient due to the increased local concentration of cell-surface receptor for cross-talking. Finally, the cell-subtype recognition and AptDzy-DNS uptake were further verified with two different cells, SK-BR-3 cells as positive group, and MDA-MB-231 cells as negative group (Fig. 5). As shown in Fig. 5, the bright fluorescence from FITC-AptDzy-DNS was only observed within SK-BR-3 cells. Only little scattered fluorescence were observed from MDA-MB-231 cells, demonstrating the specific recognition and high-efficiency delivery of AptDzy-DNS to target cancer cells.

### Internalization process of AptDzy-DNS

The colocalization experiment was carried out to confirm the integrity of AptDzy-DNS nanostructures when they crossed through cell membrane. Here, FITC and Cy3 were labeled on the different AptDzy-DNS units and the assembled AptDzy-DNS was incubated with SK-BR-3 cells for 2 h, respectively. The colocalization results demonstrated that the fluorescence from FITC and Cy3 appeared nearly in the same place (Fig. S4), further suggesting that the constructed AptDzy-DNS has the characteristics of integrity and rigidity.

### Cell cytotoxicity assay and proliferation assay

**Figure 6:**
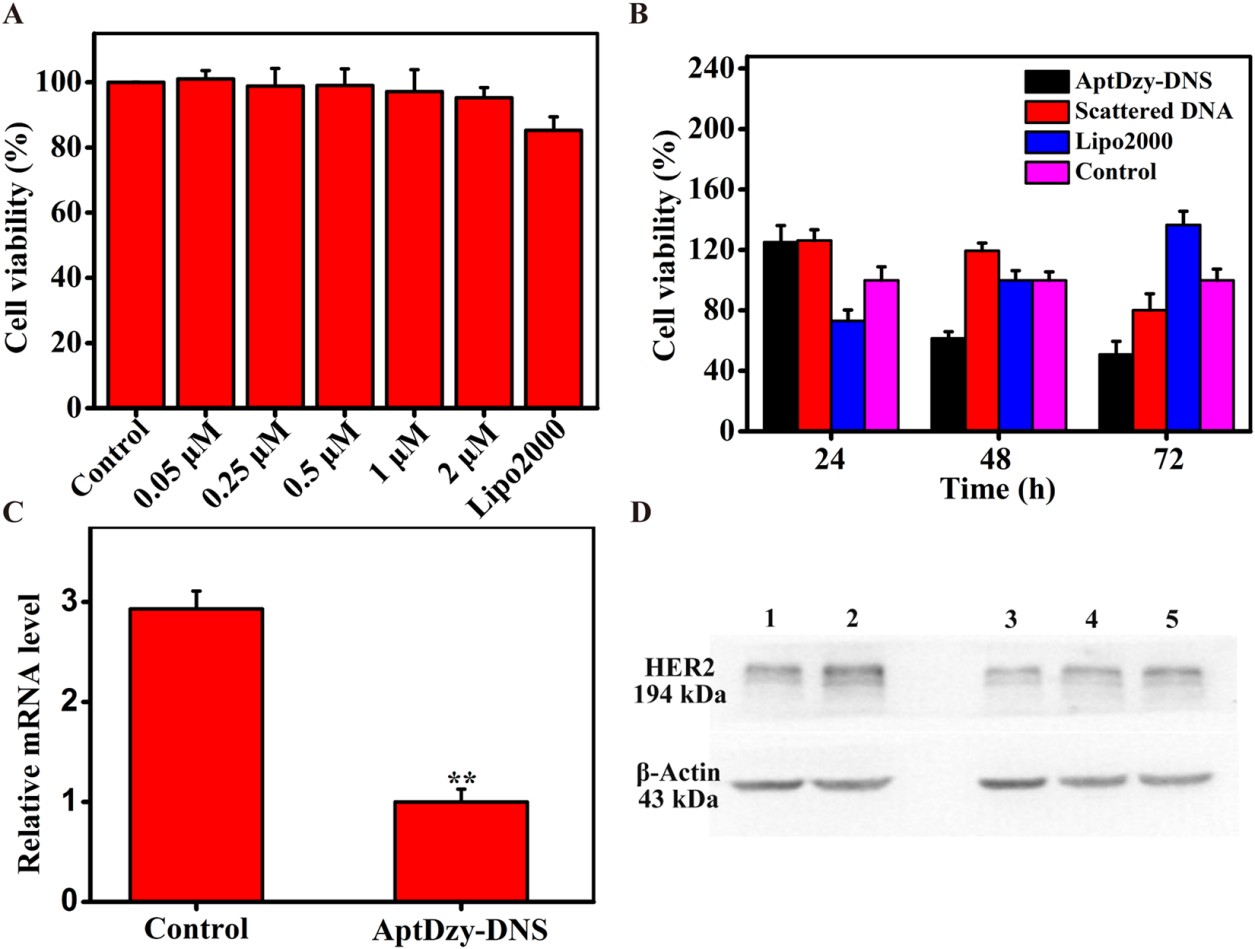
Cell cytotoxicity assay and proliferation assay. (**A**) CCK-8 assay of cell viability for SK-BR-3 cells incubated with DNS at various concentration. (**B**) CCK-8 assay of cell viability for SK-BR-3 cells incubated with AptDzy-DNS at different hours. (**C**) RT-PCR characterization of HER2 mRNA expression for SK-BR-3 cells incubated without and with AptDzy-DNS. The data error bars indicate means ± SD (n = 5). *P < 0.05, **P < 0.01 (two-tailed Student’s t-test), (**D**) Western blot characterization of HER2 expression for SK-BR-3 cells incubated with 0.25 (Lane 1), 0 (Lane 2), 0.5 (Lane 3), 0.25 (Lane 4), and 0.125 (Lane 5) μM AptDzy-DNS.

The cytotoxicity of DNS was evaluated using a cell counting kit-8 (CCK-8) assay with a series of DNS concentrations and the standard transfection reagent, Lipofectamine 2000 (Lipo2000). The cells only treated with PBS were selected as control group. As shown in Fig. 6A, even at a high concentration of 2 μM, cells treated with DNS (95.3%) still kept a better viability than cells treated with Lipo 2000 (85.3%). These results showed the good biocompatibility and non-cytotoxicity of DNS. The cell proliferation was also evaluated with CCK-8 assay to verify the inhibition effect of AptDzy-DNS to SK-BR-3 cells. The SK-BR-3 cells used as test groups were treated with AptDzy-DNS, scattered DNA, and Lipo2000, respectively. Next, all the cells of test groups were incubated for 24 h, 48 h and 72 h, respectively. As shown in Fig. 6B, the inhibition effect of cells treated with AptDzy-DNS, scattered DNA, and Lipo2000 were 61.5%, 119.5%, 100% at 48 h and 50.9%, 80.1%, 136.6% at 72 h, respectively. Compared with cells treated with scattered DNA and Lipo2000, cells treated with AptDzy-DNS (61.5% at 48 h, 50.9% at 72 h) demonstrated significant inhibition effect. These results demonstrated the feasibility of AptDzy-DNS as a promising alternative for targeted gene therapy.

### Gene silencing assay

To evaluate the gene silencing effect of AptDzy-DNS, real-time PCR (RT-PCR) assay and western blot was carried out to determine *HER-2* mRNA and HER-2 protein expression levels after the SK-BR-3 cells incubated with 0.5 μM AptDzy-DNS for 48 h. As observed in Fig. 6C, SK-BR-3 cells treated with AptDzy-DNS showed the enhanced inhibition of *HER-2* mRNA expression compared with control cells (*P* < 0.01). As illustrated in Fig. 6D, the HER-2 protein expression percentage was also decreased compared with cells treated with aptamer-free AptDzy-DNS (lane 1 vs. lane 2). In addition, SK-BR-3 cells incubated with 0.5 μM AptDzy-DNS (lane 3) showed the enhanced protein inhibition than that of 0.25 μM and 0.125 μM AptDzy-DNS (lane 4 and lane 5). These results suggested that the AptDzy-DNS owns the capacities of gene silence for highly efficient cancer targeted therapy.

## Conclusion

In summary, we design a targeted delivery system for precise gene therapy by multifunctional AptDzy-DNS with specific aptamers as “scorpion stingers” for targeting tumor cell and DNAzymes as “scorpion pincers” for cleaving mRNA into fragments. The as-prepared AptDzy-DNS with good biocompatibility can effectively discriminate cancer cells from normal cells by specific cross-talking between aptamers on AptDzy-DNS and overexpressed cell-surface receptors. The results demonstrate that the multifunctional AptDzy-DNS shows a promising prospect for targeted cancer cell distinguish, highly efficient gene therapy, and inhibition of cancer cell growth. The rigid structure of AptDzy-DNS with defined geometry (both length and diameter) is proved to be a simple and highly efficient platform for therapeutic reagents delivery and targeted gene therapy. In addition, the AptDzy-DNS can be used as a universal delivery vehicle for precise targeted therapies, and can be conveniently functionalized and extended as a robust strategy for specific precise delivery of other functional nucleic acids or DNA-binding proteins. Thus, this developed strategy provides impressive improvement on gene targeted therapies and paves the way for application of AptDzy-DNS in human cancer targeted therapies.

## Experiment Section

### Materials and Reagents

All DNA oligonucleotides (Supporting Information, Table S1) were synthesized and purified by Sangon (Shanghai, China). The DNA oligonucleotides were dissolved in 1 × Tank buffer (20 mM Tris,125 mM NaCl, 20 mM KCl, pH 7.5) and diluted in appropriate buffer prior to use or stored at −20 °C. DNA Marker, GoldView, SYBR Premix Ex TaqTM II and PrimescriptTM RT reagent Kit with gDNA Eraser were purchased from TaKaRa (Dalian, China). Dulbecco’s Modified Eagle Medium (DMEM) and Fetal Bovine Serum (FBS) were obtained from Gibco (Shanghai, China). CCK-8 assay was obtained from Dojindo (Shanghai, China). Phospho-HER2/ErbB2 Antibody Sampler Kit and β-Actin polyclonal antibody were obtained from Cell Signaling Technology (Shanghai, China). HRP anti-mouse and rabbit IgG were from Beyotime (Jiangsu, China).New Super ECL Assay was from KeyGen (Nanjing, China). RIPA (50 mM Tris (pH 7.4), 150 mM NaCl, 1% Triton x-100, 1% sodium deoxycholate, 0.1% SDS, sodium orthovanadate, sodium fluoride, EDTA, and leupeptin), phenylmethanesulfonyl fluoride (PMSF) and Bradford protein dye reagent were used in western blot. SK-BR-3and MDA-MB-231 (the human breast cancer cell lines) cells were obtained from the American Type Culture Collection (ATCC) (Rockville, MD, USA). All other reagents were of analytical grade, and Millipore-Q water (≥18 МΩ) was used in all experiments.

### Apparatus

The gel electrophoresis was performed on Second-Dimension Mini Format Electrophoresis Systems (Bio-Rad, USA) and imaged on a Bio-Rad ChemDoc XRS imager (Bio-Rad, USA). Atomic force images were performed on a Bruker Atomic Force Microscope (Germany). Fluorescence signal was recorded by an Agilent Cary Eclipse fluorescence spectrophotometer (USA). Immunofluorescence and confocal imaging were performed on a NikonA1R Confocal Microscope (Japan). CCK-8 assay was measured by a BioTek microplate reader (USA). The concentration of all RNA and DNA oligonucleotides were measured by a Thermo Nano Drop™ 1000 Spectrophotometer (USA). The RT-PCR was performed on a QIAGEN Rotor-Gene Q (Germany). The WB images were performed on a VIAGENE enhanced chemiluminescence (ECL) ion system (USA).

### Preparation of AptDzy-DNS

The multifunctional AptDzy-DNS with two overhangs of DNAzymes at each long arm was synthesized and improved according to our previous work^39^. In particular, the multifunctional AptDzy-DNS was annealed with 0.8 μM of DNA strands 1–9 and 10 (set strand) (Fig. 1A) in 1 × TAE/Mg^2+^ buffer (40 mM Tris, 20 mM acetic acid, 2 mM EDTA and 7.5 mM magnesium acetate, 20 mM potassium acetate, pH 8.0). All samples were annealed in a T100TM Thermal Cycler (Bio-Rad, U.S.A.). The temperature steps were described in Table S2.

### Cell culture and AptDzy-DNS induced translocation

SK-BR-3 and MDA-MB-231 cells were cultured in DMEM supplemented with 10% FBS, penicillin (100 U/ml) and streptomycin (100 µg/ml) at 37 °C in a humidified incubator (5% CO_2_ and 95% air). For AptDzy-DNS translocation, SK-BR-3 cells were firstly seeded in cell culture dish or well plates for 12 h. Following, cells were washed with PBS and incubated with AptDzy-DNS at a final concentration of 0.5 μM for an appropriate length of time.

### CCK-8 assay

CCK-8 assay was used to evaluate the cell cytotoxicity and proliferation of DNA nanostructures. SK-BR-3 (3 × 10^3^ per well) cells were seeded into 96-well plates for 12 h. Subsequently, cells were washed with PBS and incubated with serial concentrations of the AptDzy-DNS for an appropriate length of time in the incubator. After that, the cells were washed twice with PBS buffer, added 10 μL of CCK-8 solution and incubated for 4 h. And then the optical density was measured at a wavelength of 450 nm with a microplate reader. The relative cell viability (%) was calculated by (Atest/Acontrol) × 100.

### Western blot analysis

Western blot was used for monitoring protein expression in cells. Cells were washed with ice-cold PBS (pH 7.4) and cleaved by lysis solution (RIPA: PMSF = 1:100). Protein concentration was measured with Bradford protein dye reagent by a microplate reader to ensure the equal amount of proteins when loading samples. The proteins were separated by 8% SDS-PAGE before blotted onto polyvinylidene fluoride (PVDF) membrane. And then the membrane was blocked with 5% fat-free milk and incubated with primary antibodies (diluted at ratios of 1:1,000 (β-Actin, bioworld #AP0060; Phospho-HER2/ErbB2 Antibody Sampler Kit, CST #9923)) at 4 °C overnight or 25 °C for 2 h. The complex of antigen-antibody was visualized with New Super ECL Assay and detected by an ECLion system.

### RT-PCR analysis

Total cellular RNA was extracted with TRIzol reagent (Invitrogen, Canada) and was reverse transcribed into cDNA. RT-PCR was performed using the SYBR Premix Ex TaqTM II. All reactions were in a 25 μL reaction volume in triplicate. PCR conditions: initial denaturation at 95 °C for 30 s, 40 cycles of PCR amplification at 95 °C for 5 s, 59 °C for 30 s and 72 °C for 20 s. The relative amount of *HER2* gene mRNA was normalized to β-actin with 2^−∆∆t^ method.

## Electronic supplementary material

Supplementary Information
