# Supplementary material for: A multifunctional DNA nano-scorpion for highly efficient targeted delivery of mRNA therapeutics
Source: Sci Rep. 2018 Jul 5;8:10196. doi: 10.1038/s41598-018-28542-3 (PMC6033943; doi:10.1038/s41598-018-28542-3)
Supplement: Supplementary file 1 — Supplementary Information [file 41598_2018_28542_MOESM1_ESM.doc]

Electronic Supplementary Information for

A multifunctional DNA nano-scorpion for highly efficient targeted delivery of mRNA therapeutics

Dandan Li^1,*^, Fei Mo^2,*^, Jiangling Wu^2^, Yong Huang^1^, Huihao Zhou^1^, Shijia Ding^2^ & Weixian Chen^1^

^1^Department of Laboratory Medicine, The Second Affiliated Hospital of Chongqing Medical University, Chongqing 400010, P.R. China.

^2^Key Laboratory of Clinical Laboratory Diagnostics (Ministry of Education), College of Laboratory Medicine, Chongqing Medical University, Chongqing 400016, P.R. China.

^*^These authors contributed equally to this work.

Correspondence and requests for materials should be addressed to S.D. and W.C. (email: [dingshijia@163.com](mailto:dingshijia@163.com) and chenweixian75@163.com.)

**Supporting tables**

**Table S1** Sequences of the used oligonucleotides

| Strand number^a^ | Sequence (5’- 3’) |
| --- | --- |
| 1 | TTTTCGACCGAGCGTGAATTAGTGATCCGGAACTCGCGCAATGAACC |
| 2 | TTTTCAGCTGGCCTATCTAAGACTGAACTCGCACCGCCGGCATAAGCTATGCGCTCTGCCGC |
| 3 | TTTTAGGAGATGGCACGTTAATGAATAGTCTCCACTTGCATCCGAGATCCGAACTGCTGCCTTTT |
| 4 | TTTTCGAGAGAAGGCTTGCCAGGTTACGTTCGTACATCGTCTGAGTTTTTT |
| 5-NApt | GGTGGTGGTGGTTGTGGTGGTGGTGGGGCAGCAGTTCAGGCCAGCTGA |
| 5-HApt | GGCAGCAGTTCAGGCCAGCTGAGCAGCGGTGTGGGGGCAGCGGTGTGGGGGCAGCGGTGTGGGG |
| 6 | TTTTGGTTCATTGCGGAGTTCAGTCTTAGATGGATCTCGGATGCAAGGCCTTCTCTCGTTTT |
| 7 | GGTGCCGAGTTCCGGATCACTAATTCCATAGCTTATGCCGGCAAAGCGTAAGACCCACAATCGCAAAACTATTCATTAACGTGTGTACGAACGTAACCTGGCAATGGAG |
| 8 | TTTTGCGGCAGAGCGACGCTCGGTCGTTTTAGATGCCAGGCTAGCTACAACGATGTGGTTGA |
| 9 | AACTCAGACGACCATCTCCTAATTTTACCATTGTTCCGAGCCGGTCGAAAGCACGGCCT |
| 1’-Cy3 | Cy3 TCGACCGAGCGTGAATTAGTGATCCGGAACTCGCGCAATGAACC |
| 4’-FITC | FITC CGAGAGAAGGCTTGCCAGGTTACGTTCGTACATCGTCTGAGTT |
| Set | CGTGTGGTTGTTTGCGATTGTGGGTCTTACGCTTT |
| mRNA 1 | FITC-TCAACCACArGrUGGCATCTG-BHQ1 |
| mRNA 2 | FITC-TAGGCCGTGCTrAGACAATGGT-BHQ1 |
| HER2-F | GGCTTCTTCTGTCCAGACCC |
| HER2-R | AAATACATCGGAGCCAGCCC |
| β-actin-F | TGACGTGGACATCCGCAAAG |
| β-actin-R | CTGGAAGGTGGACAGCGAGG |

^a^ NApt, anti-nucleolin aptamer; HApt, anti-HER2 aptamer; mRNA, messenger RNA, F, forward; R, reverse.

**Table S2** Thermal annealing program for assembly of opened DNA nanotweezer.

| Temperature (^o^C) | 95 | 90-80 | 78-68 | 65-58 | 57-48 | 47-36 | 35-30 | 29.5 | 28 | 27 | 25 | 4 |
| --- | --- | --- | --- | --- | --- | --- | --- | --- | --- | --- | --- | --- |
| Time (min) | 2 | 10 | 10 | 20 | 20 | 20 | 20 | 10 | 10 | 10 | 10 | hold |

**Supporting figures**

**
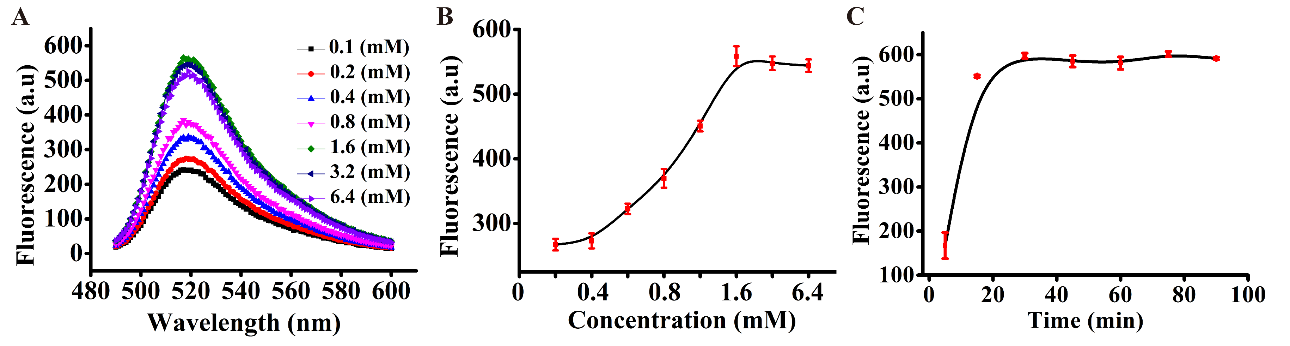
**

**Figure S1** Optimization of Mg^2+^ concentration for AptDzy-DNS digesting mRNA


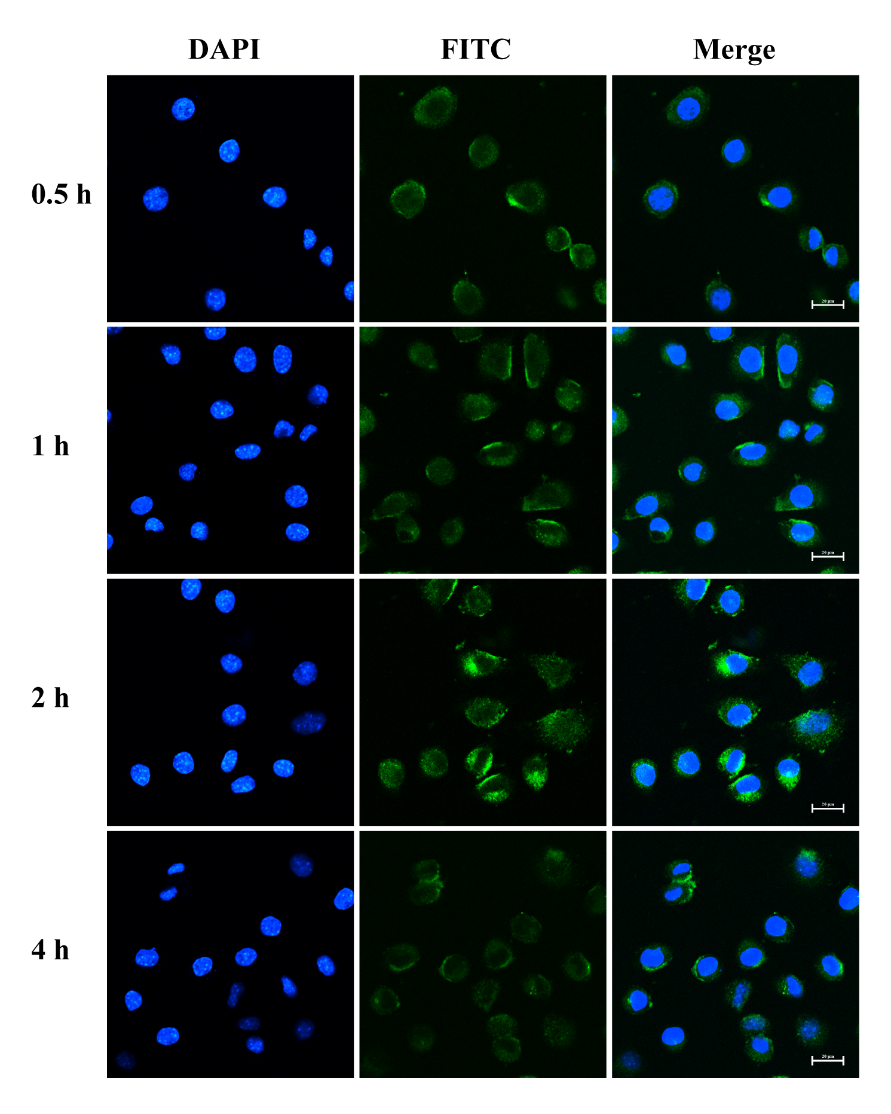


**Figure S2** Optimization of internalization time of AptDzy-DNS. (a) Confocal fluorescence microscopic images of SK-BR-3 cells after incubation with 0.5 μM incubated with AptDzy-DNS for 0.5 h, 1 h, 2 h and 4 h, respectively. The cell nucleus was stained with DAPI. The merge image shows colocalization of fluorophores in cytoplasm of SK-BR-3 cells. Scale bars: 20 μm.

**
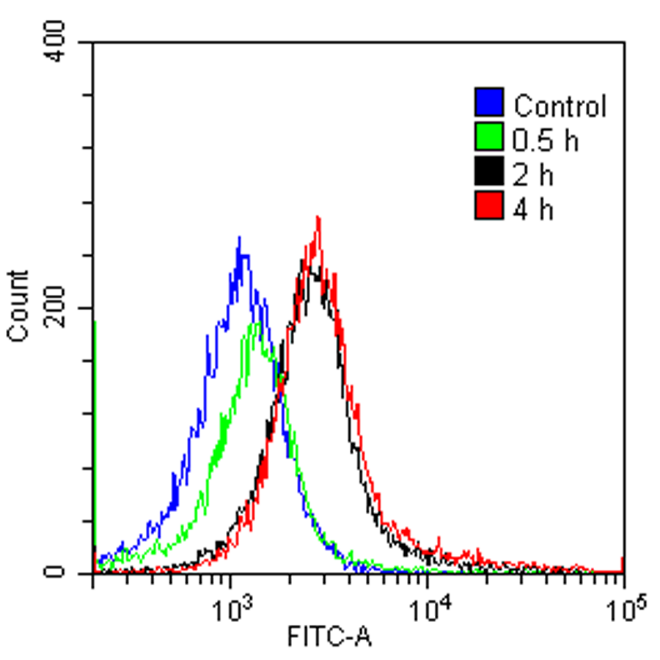
**

**Figure S3** Flow cytometry analysis of SK-BR-3 incubated with FITC-labeled AptDzy-DNS over time.


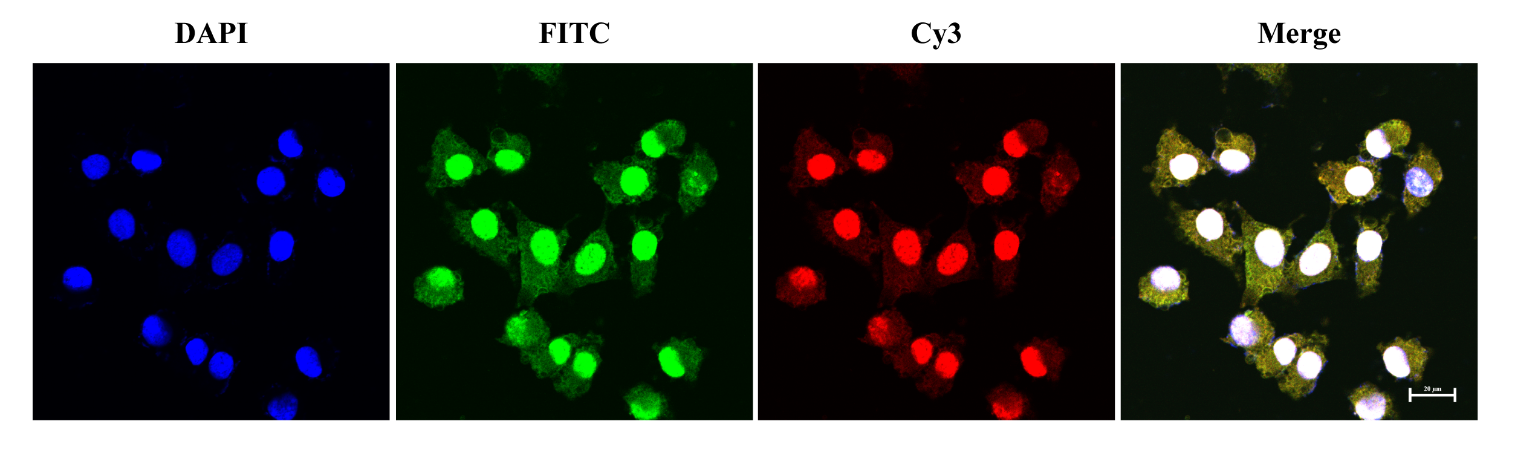


**Figure S4** The colocalization experiment for confirming the integrity of AptDzy-DNS nanostructures.
